# Pak1 Kinase Promotes Activated T Cell Trafficking by Regulating the Expression of L-Selectin and CCR7

**DOI:** 10.3389/fimmu.2019.00370

**Published:** 2019-03-05

**Authors:** Ana Dios-Esponera, Nicolas Melis, Bhagawat C. Subramanian, Roberto Weigert, Lawrence E. Samelson

**Affiliations:** Laboratory of Cellular and Molecular Biology, Center for Cancer Research, National Cancer Institute, National Institutes of Health, Bethesda, MD, United States

**Keywords:** Pak1 kinase, L-selectin, CCR7, lymph node trafficking, L-selectin shedding

## Abstract

Normal function of the adaptive immune system requires trafficking of T cells between the blood and lymphoid organs. Lymphocyte homing to lymph nodes requires that they cross endothelial barriers present in blood vessels and lymphatics. This multi-step process requires a remodeling of the lymphocyte plasma membrane, which is mediated by the dynamic re-arrangement of the actin cytoskeleton. Pak1 plays a central role in cell morphology, adhesion and migration in various cell types. Here we demonstrate that Pak1 is required for activated CD4^+^ T cell trafficking to lymph nodes. Pak1 deficiency in T cells causes a defect in the transcription of CCR7 and L-selectin, thereby altering lymphocyte trafficking. Additionally, we report an increase in L-selectin shedding in Pak1-deficient T cells, which correlates with a decrease in the recruitment of calmodulin to the cytoplasmic tail of L-selectin during T cell activation. Overall, our findings demonstrate that by regulating the expression of two major lymph node homing molecules, L-selectin and CCR7, Pak1 mediates activated CD4^+^ T cell trafficking.

## Introduction

T cell trafficking is required for normal function of the adaptive immune system. Naïve T cells continuously recirculate through blood, lymphatics, and secondary lymphoid organs (1). The process of extravasation from blood into secondary lymphoid organs is known as transendothelial migration (TEM) and is regulated by sequential steps. First, T cells roll over the vascular endothelium and then arrest or adhere to the vascular wall. Lymphocytes then cross the endothelial barrier by diapedesis (2). Several surface molecules are required for each step of the process. Rolling is mediated by interaction of selectins present in the T cell and the endothelial cell, whereas chemokine receptors and integrins regulate the adhesion, intravascular crawling, and diapedesis. Once inside the secondary lymphoid organs, naïve T cells scan antigen-presenting cells for cognate antigen and differentiate into effector T cells (1). Following the resolution of the T cell response, most effector T cells die, but some responding T cells differentiate into one of two types of memory T cells, central or effector (3). The major difference between these two subsets is that central memory T cells express lymph node homing molecules, while effector memory T cells recirculate through non-lymphoid tissues, maintaining effector-like functions. Variations in the expression of chemokine receptors and adhesion molecules mediate changes in the T cell trafficking essential for a correct immune response. Naïve and central memory T cells, express the chemokine receptor, CCR7 and the adhesion molecule, L-selectin, required for migration to lymphoid organs (4), whereas in effector and effector memory T cells CCR7 and L-selectin are downregulated, and these cells instead express tissue homing receptors such as the integrin, VLA-4, which targets the cells to non-lymphoid tissues.

The p21-activated kinases, Paks, are serine/threonine kinases involved in multiple cellular processes, including cytoskeleton remodeling and cell motility, MAPK signaling, apoptotic signaling, and they are involved in many human diseases including cancer (5). Pak1 and Pak2 are expressed in T cells, with Pak2 expression higher than Pak1 (6). Pak2 is essential for regulation of the development and maturation of thymocytes (7–9). There are three separate pathways that activate Pak1 in T cells. The first involves the activation of Vav1, a GEF for the small GTPases Rac1/Cdc42, a process dependent on the adapters proteins LAT, SLP-76, and Nck (7). The second pathway uses the adaptors GIT and PIX, which coordinate the activation of Rac1 and Cdc42 (10, 11). The third pathway involves the Pak1/PLC-γ1/Bam32 complex, which is specific for Pak1 but not Pak2 activation and it is PIX-, Nck-, and Rac1/Cdc42-independent (12). While JNK activation by Pak1 in Jurkat T cells following TCR engagement has been questioned (8), we have previously described that Pak1 activity increases JNK activation in primary T cells and in Jurkat T cells. In these settings Pak1 is involved in apoptosis in T cells mediated by the nuclear translocation and activation of the transcription factor FOXO3, which leads to BIM and caspase 9 activation with cytochrome C release (13). In addition, we have also shown a role for a trimolecular complex between PAK1, PLCγ-1, and Bam32 in ERK activation in CD4^+^ T cells (12).

Based on the fact that proteins implicated in cytoskeletal and morphological polarization are essential for intranodal T cell migration (14–18) and are known to act as Pak1-activators or Pak1-effectors, we sought to determine whether Pak1 might influence T cell migration and trafficking *in vivo*, and if so, by what mechanism. Here, we report for the first time that conditional deletion of Pak1 substantially affected the homing of activated CD4^+^ T cells to lymph nodes by altering the regulation of several genes involved in T cell trafficking, which include L-selectin and Ccr7. Moreover, the absence of Pak1 also down-regulates the expression of L-selectin at the surface by increasing its proteolytic cleavage and shedding. Finally, increasing the levels of L-selectin in activated Pak1-deficient T cells, produced a recovery in T cell homing to lymph nodes.

## Materials and Methods

### Mice

Mice with a conditional allele of *Pak1* (*Pak1*^fl/fl^) were kindly provided by X. Wang (Manchester University) (19). Lck-Cre (20) mice were purchased from Taconic. T cell-specific deletion of *Pak1*[denoted *Pak1*(T)^−/−^] was achieved by crossing *Pak*^fl/fl^ mice with mice expressing the distal Lck promoter (LCK-Cre). The primers used for screening Pak1 for deletion were: Pak1F- TCTTGGGAGCATCTCAGGAACATCTT and Pak1R- GCTCCTGTGAAGGGCAAGTTCTAT (WT 270 bp, Targ 310 bp). The PCR reaction was annealed at 51°C. The primers used to assess Cre activity in the tissues of interest were CreF- CCTGGAAAATGCTTCTGTCCGTTTG and CreR- ACGAACCTGGTCGAAATCAGTGCG. Mice were housed according to the guidelines of the Animal Care and Use Committee at the National Institutes of Health (NIH). All the experiments performed were approved by the Animal Care and Use Committee of the NIH.

### Cell Culture

Naïve purified CD4^+^ and CD8^+^ T cells were isolated as previously described (21). Briefly, cells were purified from lymph nodes or spleen by negative selection (90–98%) (Stem Cell Technology) according to the manufacturer's specifications. T cell blasts were obtained after stimulation for 3 days on plate-bound anti-CD3ε (2 μg/mL) (BD Biosciences), with added soluble anti-CD28 (1 μg/mL) (BD Biosciences), and IL-2 (100 U/mL) (NCI Biological Resource Branch) in regular RPMI medium (supplemented with 10% fetal bovine serum, 1% penicillin-streptomycin and 50 μM beta-mercaptoethanol). Then T cell blasts were expanded with IL-2 (100 U/mL) for 2 more days and starved of IL-2 for 24 h (resting phase).

### *In vivo* Homing

C57BL/6 mice were injected intravenously as previously described (21). Briefly, a 1:1 mixture of splenocytes (10 x 10^6^ cells in total) from WT and *Pak1*(T)^−/−^ mice, labeled with 1 μM CellTracker Green CMFDA Dye (Invitrogen) or 1 μM Cell Trace Violet Stain (Invitrogen) were injected into recipient mice. Dyes were swapped in repeat experiments. After 1 h, recipient mice were euthanized, and blood, spleen, and lymph nodes were harvested, stained with an anti-CD4 or anti-L-selectin (MEL-14) antibodies and analyzed by flow cytometry to determine the ratio between CMFDA- and Cell Trace Violet-labeled CD4^+^ T cells.

CD4^+^ T cell blasts from WT or *Pak1*(T)^−/−^ mice were loaded with Cell Trace Violet or Cell Trace Far Red (Invitrogen). Cells were mixed at a ratio of 1:1, and 10 x 10^6^ cells were injected into the tail veins of C57BL/6 mice. One hour after injection, recipient mice were euthanized, and blood, spleen, and lymph nodes were removed for quantification of Cell Trace Violet-labeled and Cell Trace Far Red-labeled T cells by flow cytometry.

For mTOR inhibitor rapamycin experiments, CD4^+^ T cell blasts were cultured with IL-2 (100 U/mL) in the presence or absence of 20 nM of rapamycin (Sigma-Aldrich) for 48 h. Cells were loaded with Cell Trace Violet, CMFDA or FarRed Dyes, mixed equally, and 10 × 10^6^ cells were injected into the tail vein of C57BL/6 mice. 1 h later, mice were sacrificed, and blood, spleen, and lymph nodes were removed for analysis.

Cell acquisition was performed on an LSRFortessa (BD Biosciences) flow cytometer. Data analysis was performed using FlowJo software (Tree Star, Inc., Ashland, OR, USA). The number of recovered CD4^+^ T cells was expressed as a percentage of the total number of injected cells.

### Two-Photon Intravital Microscopy

Inguinal lymph nodes were prepared for intravital microscopy as described in (22). Briefly, 6- to 10- wk-old C57BL/6 mice were anesthetized by an i.p. injection of a mixture of Ketamine, Acepromazine and Xylazine (80, 2, and 4 mg.kg^−1^, respectively). The right LN was surgically exposed and freed from connective and adipose tissue. Special care was taken to avoid damage to blood vessels and afferent lymphatic vessels during surgery. HEVs and blood vessels were labeled by retro-orbital injection of anti-mouse PNAd (MECA-79) (BD Biosciences) conjugated with Pacific Blue antibody (Invitrogen) and Evans Blue (Sigma-Aldrich), respectively. The mice were placed on a 37°C warmed cover-glass chamber slide filled with PBS. Prior to adoptive transfer, the surgically exposed inguinal LN was imaged and then 10 × 10^6^ CD4^+^ T cell blasts purified from either WT or *Pak1*(T)^−/−^ mice were fluorescently labeled with 1 μM CMFDA or 2 μM CellTracker Red CMTPX (Invitrogen) and were adoptively transferred by venous injection into the recipient mice.

For experiments with the ADAM17 inhibitor TMI-1, CD4^+^ T cell blasts were cultured with anti-CD3ε (2 μg/mL), anti-CD28 (1 μg/mL) and IL-2 (100 U/mL) for 3 days, and then with IL-2 (100 U/mL) for 2 more days in the presence or absence of 10 μM of TMI-1 (Tocris Bioscience). Cells were loaded with 1 μM CMFDA or 2 μM CellTracker Red CMTPX, mixed equally, and 10 × 10^6^ cells were injected into the tail vein of C57BL/6 mice.

Imaging was performed by using an inverted laser-scanning two-photon microscope (MPE-RS, Olympus, Center Valley, PA, USA) equipped with a tunable laser (Insight DS+, Spectra Physics, Santa Clara, CA, USA). Excitation was performed at 810 nm and the emitted light was collected by an appropriate set of mirrors and filters on 4 detectors (bandpass filters: Blue = 410–460 nm, Green = 495–540 nm, Red = 575–645 nm, and Far red = 660–770 nm). All images were acquired using a 37°C heated 25× water immersion objective NA 1.05 (XLPLN25XWMP2, Olympus).

### 3D Immunohistology

After intravital imaging, to preserve integrity of the tissue, the lymph nodes were fixed by intracardiac perfusion using a solution containing 4% formaldehyde in PBS as previously described (23). After fixation, lymph nodes were collected and prepared for the tissue clearing process: after 1 h of incubation in a solution containing 50 mM of NH_4_Cl, and 5% of FBS, lymph nodes were rinsed in PBS three times for 5 min and then incubated overnight in PBS + 1% triton X-100. After three rinses in PBS, LN were incubated for 6–10 h in FocusClear (CelExplorer), a tissue clearing solution. LN were then mounted on a coverslip and imaged with the same parameters previously used for intravital imaging.

### Intravital Microscopy Data Analysis

Imaris (Bitplane) was used for four-dimensional image analysis and automated T cell tracking. Only tracks with durations of >60 s were included in the analysis to manually control automated tracking. The parameters, average track velocity, the meandering index and the arrest coefficient were calculated using Imaris. The cutoff for arrest coefficient calculation was set to 4 μm/min.

### Flow Cytometry

Flow cytometry was performed as previously described (21). Antibodies for T cell stimulation and flow cytometry assays binding the following proteins were obtained from BD Bioscience: PE-CCR7 (4B12), APC-CD4 (RM4-5 or GKL-5), PerCP-CD8 (53-6.7), PE-CD11a (M17/4), and PE-L-selectin (MEL-14). Cell acquisition was performed on a FACSCalibur (Becton Dickinson, Franklin Lakes, NJ, USA) or an LSRFortessa (BD Biosciences) flow cytometers. Data analysis was performed using FlowJo software (Tree Star, Inc., Ashland, OR, USA).

### Quantitative Real-Time PCR

RNA was purified using the RNeasy RNA purification Mini Kit (Qiagen). Genomic DNA was digested with RNase-free DNase (Qiagen) following the manufacturer's instructions and reverse-transcribed using the iScript cDNA synthesis kit (BioRad). Quantitative PCR was performed in a 384-well plate format using Power SYBR Green PCR master Mix (Applied Biosystems) on an Applied Biosystems Real-Time PCR instrument. GAPDH mRNA was used for normalization.

### Primers

*Gapdh* forward: 5′-GTGGAGATTGTTGCCATCAA-3′

*Gapdh* reverse: 5′-CGTCCCGTAGACAAAATGGT-3′

*Sell* forward: 5′-ACGGGCCCCCGTGTCAGTATGTG-3′

*Sell* reverse: 5′-TGAGAAATGCCAGCCCCGAGAA-3′

*Ccr7* forward: 5′-TGATTTCTACAGCCCCCAGA-3′

*Ccr7* reverse: 5′-GCACACCTGGAAAATGACAA-3′

*Klf2* forward: 5′ -TGTGAGAAATGCCTTTGAGTTTACTG-3′

*Klf2* reverse: 5′ -CCCTTATAGAAATACAATCGGTCATAGTC-3′

### Cell Stimulation, Lysis Immunoprecipitation, and Western Blot Analysis

CD4^+^ T cell blasts were stimulated as described before (21). Briefly, cells were rested overnight in regular RPMI medium prior to activation. For TCR stimulation CD4^+^ T cells were incubated for 15 min at 4°C with biotinylated anti-CD3ε (10 μg/mL, BD Biosciences) antibodies. Cells were washed and stimulated for the indicated time by adding streptavidin (20 μg/mL final concentration). For CCR7 stimulation, rested T cell blasts were stimulated with 200 ng/mL of CCL21 (R&D Systems). After rapid centrifugation, cells were lysed at 4°C for 10 min in 1% NP-40 lysis buffer (50 nM Tris pH 7.4, 150 mM NaCl, 5 mM EDTA, protease inhibitor cocktail [Roche], 1 mM Na_3_VO_4_, 0.1% SDS). Lysates were centrifuged at 14,000 rpm for 10 min at 4°C. For immunoprecipitation, post-nuclear supernatants were pre-cleared with 4 μg of normal mouse IgG bound to 20 μL of Protein A/G Plus-Agarose beads (Santa Cruz Biotechnology) for 1 h at 4°C. The precleared samples were incubated with 4 μg of the indicated antibody previously conjugated to 30 μL Protein A/G Plus-Agarose beads. After incubation for 2 h at 4°C, the immunoprecipitates were washed three times with ice-cold lysis buffer. The immunoprecipitates were eluted with 2× Laemmli buffer (4% SDS, 10% beta-mercaptoethanol, 20% glycerol, 0.004% bromophenol blue, 0.125 M Tris-HCl), and the eluents were subjected to SDS-PAGE and transferred to nitrocellulose membranes. These blots were incubated overnight at 4°C with the corresponding primary antibody directed against either anti-phospho-AKT (Thr308) or (Ser473) (Cell Signaling Technology), anti-AKT (Cell Signaling Technology), anti-β-Actin (Sigma-Aldrich), anti-FOXO1 (Cell Signaling Technology), anti-Calmodulin (Merck-Millipore), or anti-L-selectin/L-SELECTIN (R&D systems). Blots were incubated with horseradish peroxidase–conjugated secondary antibodies (GE Healthcare) for 1 h at room temperature. ECL (enhanced chemiluminescence; SuperSignal West Pico and SuperSignal West Femto, Pierce) was used to visualize protein bands, which were quantified with ImageJ software (NIH).

### FOXO1 Subcellular Localization

Nuclear and cytoplasmic extracts were obtained from 25 × 10^6^ cells using NER-PER Nuclear and Cytoplasmic Extraction Reagents (Thermo Scientific), following the manufacter's instructions. Lamin B and beta-tubulin were used as nuclear and cytoplasmic markers, respectively.

### L-selectin Shedding Assay

The concentrations of sL-selectin released into the supernatants of blast WT and *Pak1(T)*^−/−^ CD4^+^ and CD8^+^ T cells were measured using a mouse sL-selectin/L-selectin ELISA kit according to the manufacturer's instructions (R&D Systems). The amount of soluble L-selectin present in the supernatant was calculated as pg/mL, and expressed as fold induction to sL-selectin pg/mL WT.

Where indicated, CD4^+^ T blast cells were incubated for 20 min with DMSO (1:1,000), 10 μM BAPTA-AM (Invitrogen) and 2 mM EGTA or 0.5 μM of the ADAM17 inhibitor TMI1 (Tocris Bioscience). After washing, cells were prepared at 2 × 10^6^ cells/mL in RPMI medium in the presence of DMSO (1:1,000), 2 mM EGTA, or 0.5 μM TMI1. Cells were then incubated at 37°C in 5% CO^2^ in the presence of 100 ng/mL CCL21 or 50 ng/mL PMA. After 30 min, cells were centrifuged, and supernatants were harvested for sL-selectin ELISA.

### Calcium Flux Measurement

Calcium flux was performed as previously described (24). Briefly, cells were incubated with 5 mM Indo-1 dye (Invitrogen) and 0.5 mM Probenicid (Sigma-Aldrich) in RPMI without phenol red for 45 min at 37°C. Cells were washed with RPMI, re-suspended in RPMI, and incubated at 37°C for 5 min before Ca^2+^ measurement. A baseline reading was taken for 30 s and cells were stimulated adding CCL21 (100 ng/mL). Samples were analyzed with an LSRFortessa (BD Biosciences) with a UV laser, and data were analyzed with FlowJo software. Calcium increases were monitored as the ratio of Indo-1 (blue) and (violet) emission and displayed as a function of time.

### Statistical Analysis

Graph-Pad Prism software was used for data analysis. Two-tailed *t*-tests were used to calculate statistical significance between two groups, while two-way analysis of variance or ANOVA, was used to determine the statistical differences among several groups. Data are expressed as mean ± SEM. Values of ^*^P < 0.05, ^**^P < 0.01, ^***^P < 0.001 were considered significant.

## Results

### Pak1 Is Necessary for Activated CD4^+^ T Cell Migration to Lymph Nodes

**Figure 1:**
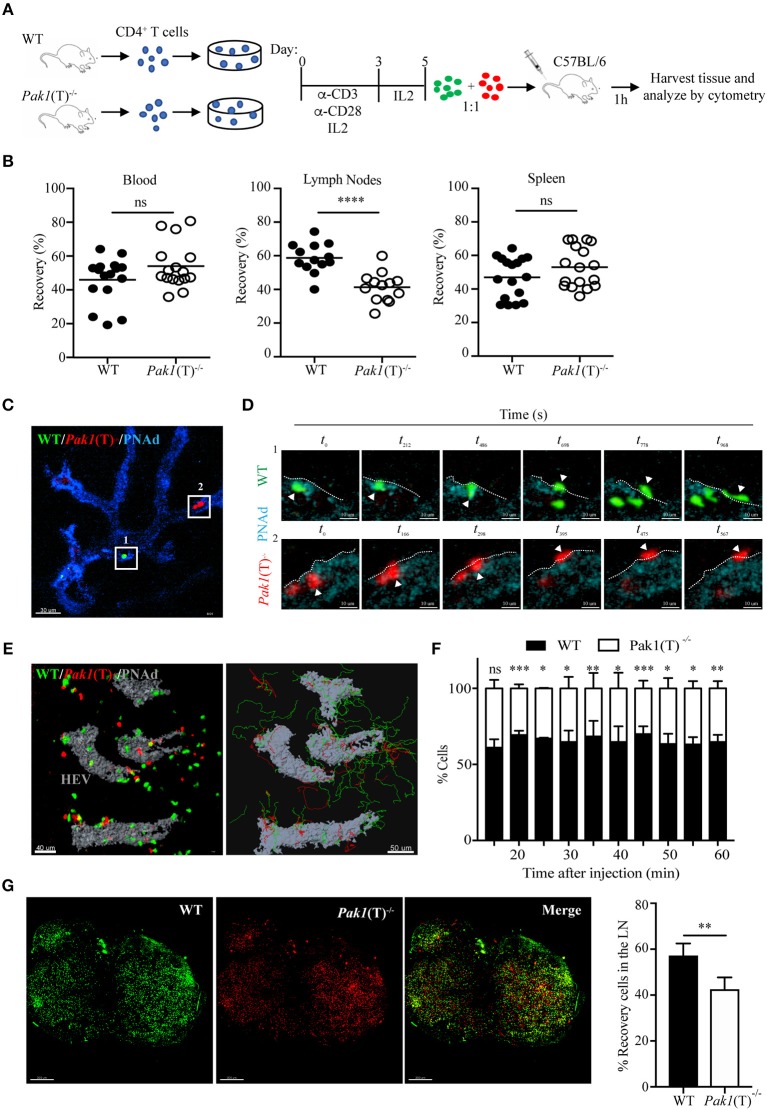
Pak1 is required for efficient homing of activated CD4^+^ T cells into lymph nodes. **(A)** Schematic of *in vitro* CD4^+^ T cell activation and co-transfer 1:1 of differentially dye-labeled WT and *Pak1*(T)^−/−^ T cell blasts in recipient C57BL/6 for T cell trafficking to lymphoid tissues by flow cytometry quantification. **(B)** Recovery of co-transferred labeled WT or *Pak1*(T)^−/−^ cells in A, presented as a percentage of total transferred cells recovered from the blood, LNs or spleen at 1 h after transfer. Each dot indicates result from an individual mouse; horizontal bars indicate the mean. Data were pooled from four to five recipient mice of three to four independent experiments. Sample sizes: 21, blood and spleen; 19 lymph nodes. **(C)** 2-photon intravital image of the HEV at 15 min after transfer of WT, green; and *Pak1*(T)^−/−^, red; CD4^+^ T cell blasts. HEV are visualized with an anti-PNAd-Pacific blue antibody (Blue). Box 1 and box 2 indicate a region with a WT and a *Pak1*(T)^−/−^ CD4^+^ T cell, respectively, prior to their extravasation. Scale bar, 40 μm. **(D)** Panels show zoomed-in views of WT (green cells top, Box 1) and *Pak1*(T)^−/−^ (red cells bottom, Box 2) CD4^+^ T cell blasts moving from the inside of the vessel to the outside. *t*_0_ indicates the first time the cell adhered to the wall of the HEVs. White arrows indicate extravasating cells. White dashed lines indicate the vessel wall. Scale bars, 6 μm. **(E)** Left, 2-photon intravital image 2 h after WT and *Pak1*(T)^−/−^ CD4^+^ T cell blast transfer. HEV are labeled in gray. Scale bar, 40 μm. Right, automated tracking of CD4^+^ T lymphocytes migration. Trajectories of WT CD4^+^ T cell blasts are displayed as green tracks while *Pak1*(T)^−/−^ CD4^+^ T cell blasts are displayed in red. Scale bar, 50 μm. **(F)** Analysis of cell populations in the inguinal node at various time points from 15 to 60 min after cell transfer into the tail vein. Percentage of each population of the total is shown. Pooled from three independent experiments. **(G)** Inguinal lymph nodes explanted 2 h after adoptive transfer of dye-labeled WT and *Pak1*(T)^−/−^ CD4^+^ blast T cells were cleared following the tissue clearing process using FocusClear for 10 h. After clearing, lymph nodes were imaged on a 2-photon microscope. The panels show a 3D volume rendering image of individual dye-labeled WT (Green dye) and *Pak1*(T)^−/−^ (Red dye) CD4^+^ blast T cells and a merge. Right, quantification of dye-labeled WT and *Pak1*(T)^−/−^ CD4^+^ blast T cells in inguinal lymph nodes subjected to tissue clearing. Percentage of each population of the total is shown. Pooled from five LNs of three independent experiments. Scale bars, 250 μm. Statistical analysis: unpaired Student's *t*-test **(B,F,G)**. *ns*, not significant; *P < 0.05; **P < 0.01; ***P < 0.001; ****P < 0.0001.

To determine if the deletion of Pak1 alters T cell trafficking *in vivo*, we compared the ability of *Pak1*(T)^−/−^ and WT CD4^+^ T cells to home to secondary lymphoid organs using adoptive transfer experiments. Non-activated splenocytes from *Pak1*(T)^−/−^ and WT mice were labeled with two different cytosolic dyes. Cells were mixed at 1:1 ratio and transferred by tail vein injection into C57BL/6 host mice. After 1 h, host mouse blood, lymph nodes, and spleen cells were harvested, incubated with anti-CD4-APC antibodies, and analyzed for the presence of CD4^+^ transferred cells. Naïve *Pak1*(T)^−/−^ and WT CD4^+^ T cells showed equivalent homing to the spleen and lymph nodes after 1 h of intravenous adoptive transfer (Figure S1A). However, when CD4^+^ T cells from *Pak1*(T)^−/−^ and WT mice were first stimulated *in vitro*, and then transferred as blasts to host mice (Figure 1A), we observed a significant defect in the migration of these CD4^+^ *Pak1*(T)^−/−^ blast T cells into lymph nodes compared with WT CD4^+^ T cell blasts (Figure 1B). On the other hand, the percentage of CD4^+^ T cells that migrated to spleen or remained in the blood was similar between CD4^+^ *Pak1*(T)^−/−^ and WT T cell blasts. These results reveal a role for Pak1 in T cell migration and trafficking of activated, but not naïve T cells to peripheral lymph nodes.

To determine whether *Pak1*(T)^−/−^ T cell blasts have migratory defects, we visualized the behavior of WT and *Pak1*(T)^−/−^ T cell blasts adoptively transferred into C57BL/6 host mice. We used 2-photon intravital microscopy to follow their movement within the lumen of the high endothelial venules (HEVs) and into the parenchyma of the inguinal node (Video S1). The process of TEM was not affected by the absence of Pak1. Once WT and *Pak1* (T)^−/−^ blast T cells firmly adhered to the endothelium, the average times to find a TEM site and transmigrate did not differ (Videos S2, S3; Figures 1C,D; Figure S1B). Once CD4^+^ T cells crossed HEVs they rapidly migrated away from the cortical ridge region to enter the deep lymph node cortex (Figure 1E; Video S4). Imaging at various time points following the adoptive i.v. transfer revealed fewer *Pak1* (T)^−/−^ blast T cells localized in the inguinal node, compared with WT T cells (Figure 1F). Quantitative analysis by automated cell tracking showed no differences in velocity or in the arrest coefficient (Figures S1C,D). However, a minor difference was observed in the meandering index, with a reduced propensity of *Pak1* (T)^−/−^ blast T cells to move away from their relative starting positions in comparison with WT (Figure S1E).

A 3D imaging of cleared tissue allows examination of labeled cells in the entire lymph node. Peripheral lymph nodes isolated after 2 h of intravenous adoptive transfer of labeled CD4^+^ WT and *Pak1*(T)^−/−^ T cell blasts were analyzed using 2-photon intravital microscopy after clearing tissue. We observed an increase in the number of WT blast T cells (Green Dye) in the lymph node, compared with *Pak1* (T)^−/−^ blast T cells (Red Dye) (Figure 1G). Taken together, these data using live and fixed-cell analysis indicate that the defective lymph node homing of Pak1-deficient T cell blasts is not caused by a defect in the TEM process. Instead, there is a defect in the number of *Pak1* (T)^−/−^ blast T cells that migrate inside the lymph nodes.

### L-selectin and CCR7 Transcription in Activated T Cells Are Regulated by Pak1

**Figure 2:**
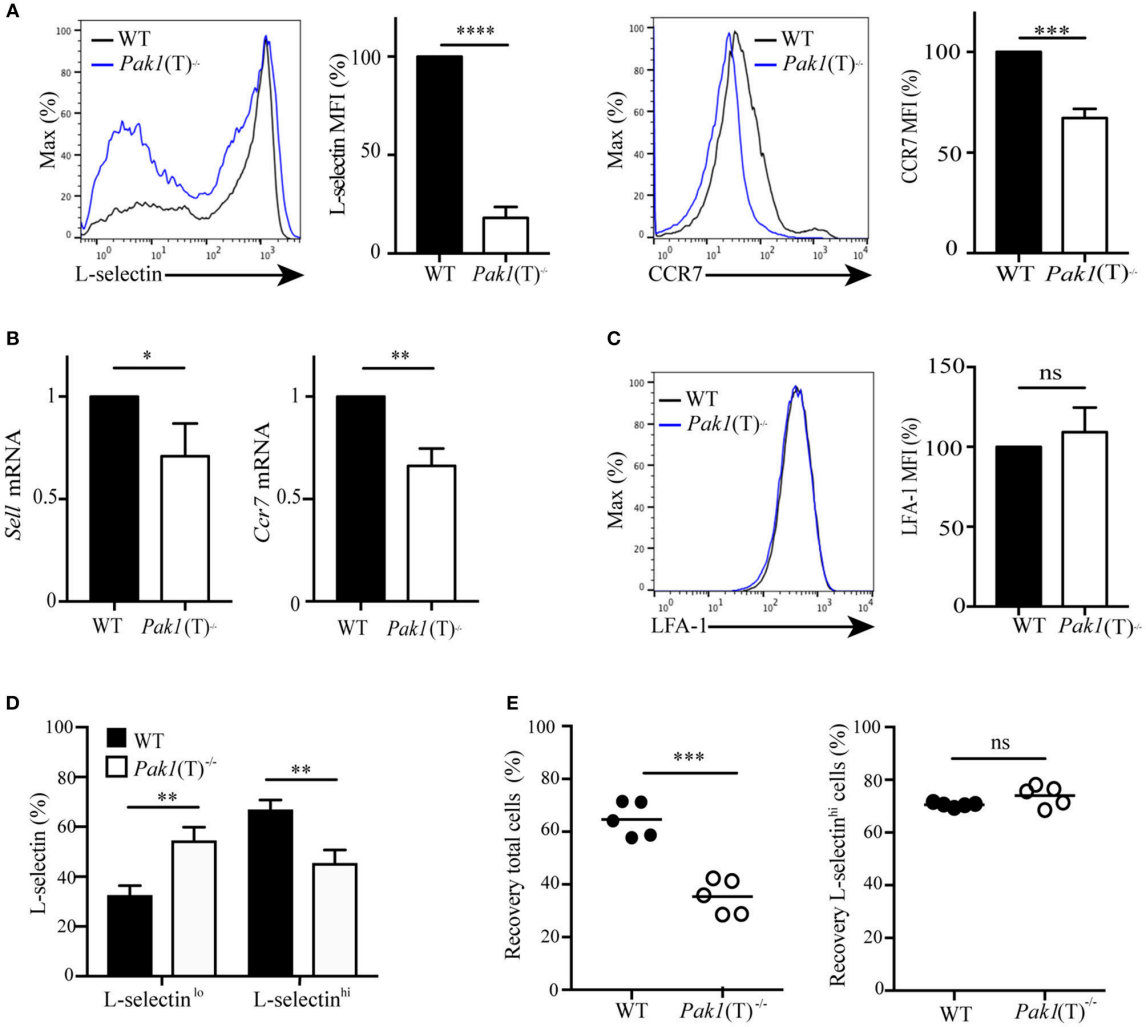
Pak1 regulates L-selectin and CCR7 transcription in activated CD4^+^ T cells. **(A**) Left, L-selectin surface marker expression as measured by flow cytometry of blast T cells. Representative histograms from eight independent experiments showing a comparison of L-selectin expression between WT and *Pak1*(T)^−/−^ are shown. Bar chart shows MFI percentage, mean fluorescence intensity, of L-selectin. Right, CCR7 surface marker expression as measured by flow cytometry in CD4^+^ T cell blasts from WT and *Pak1*(T)^−/−^ mice. The results are representative of four experiments. Bar chart shows MFI (%) of CCR7 in WT and *Pak1*(T)^−/−^ blast T cells. **(B)** Relative expression of *Sell* and *Ccr7* mRNA by qRT-PCR in CD4^+^ T cell blasts from WT and *Pak1*(T)^−/−^ mice. Pooled from six independent experiments. **(C)** LFA-1 surface marker expression as measured by flow cytometry in CD4^+^ T cell blasts from WT and *Pak1*(T)^−/−^ mice. LFA-1 expression bar chart shows MFI (%) of LFA-1 from three experiments. **(D)** Quantification of the percentage of L-selectin-negative cells (L-selectin^lo^) and L-selectin-positive cells (L-selectin^hi^) in **(A)**. The results are representative of six experiments. **(E)** Differentially dye-labeled CD4^+^ WT or *Pak1*(T)^−/−^ blast T cells were co-adaptively transferred intravenously at a 1:1 ratio, and T cell trafficking to lymph nodes was quantified by flow cytometry. Recovery of co-transferred WT or *Pak1*(T)^−/−^ blast T cells, (left) presented as a percentage of total transferred cells, or (right) presented as a percentage of L-selectin^hi^ expressing cells recovered from the LNs at 1 h after transfer. Each dot indicates result from an individual mouse; horizontal bars indicate the mean. Data were pooled from five recipient mice. Statistical analysis: unpaired Student's *t*-test **(A–C,E)** ANOVA between selected columns with Sidak's multiple comparison test **(D)**. *ns*, not significant; *P < 0.05; **P < 0.01; ***P < 0.001; ****P < 0.0001.

The chemokine receptor, CCR7, the adhesion molecule L-selectin and the integrin LFA-1 coordinate the entry of T lymphocytes in lymph nodes (2). Because CCR7-; L-selectin- and LFA-1-deficient mice have a reduction in the number of lymphocytes localized in the peripheral lymph nodes, but not in the spleen (25–27), we tested the expression of CCR7, L-selectin, and LFA-1 in naïve and T cell blasts. CCR7, L-selectin and LFA-1 surface expression was similar in naïve T cells from *Pak1*(T)^−/−^ and WT mice (unpublished data). However, in T cell blasts, analysis of L-selectin surface expression revealed that *Pak1*(T)^−/−^ CD4^+^ (Figure 2A) and CD8^+^ (Figure S2A) T cell blasts expressed less L-selectin at the surface than WT T cells. This reduction in protein expression is associated with a reduction in *Sell* mRNA (L-selectin) in both CD4^+^ (Figure 2B) and CD8^+^ (Figure S2B) T cell blasts. In addition, Pak1 deficiency also affected both protein and mRNA expression of CCR7 in CD4^+^ T cells (Figures 2A,B). However, LFA-1 expression was unaffected in *Pak1*(T)^−/−^ compared with WT CD4^+^blast T cells (Figure 2C). Our data thus indicate that the decreased trafficking of *Pak1*(T)^−/−^ T cell blasts to lymph nodes correlates with a decrease in the expression of L-selectin and CCR7.

Evaluation of L-selectin surface expression (Figure 2A) revealed an increase in L-selectin-negative cells and a decrease in L-selectin-high cells in CD4^+^ *Pak1*(T)^−/−^ blast T cells compared with CD4^+^ WT blast T cells (Figure 2D). Because L-selectin is important in the homing of T cells to peripheral lymph nodes (27), we hypothesized that the decrease in migration to lymph nodes that we observed in CD4^+^ *Pak1*(T)^−/−^ blast T cells, could be due to the downregulation in L-selectin in those cells. We transferred WT and *Pak1*(T)^−/−^ blast T cells as described in Figure 1A, and the lymph node cells were harvested 1 h later. These cells were then incubated with anti-L-selectin antibodies to analyze for the presence of L-selectin-high transferred cells. We observed a significant defect in the migration of CD4^+^ *Pak1*(T)^−/−^ blast T cells into lymph nodes compared with WT CD4^+^ T cell blasts (Figure 2E). However, of the CD4^+^ *Pak1*(T)^−/−^ blast T cells that migrated to the lymph nodes, the percentage of CD4^+^ *Pak1*(T)^−/−^ L-selectin-high T cells was similar to the percentage of CD4^+^ WT L-selectin-high T cells. This result confirms that the defect in homing of activated Pak1-deficient T cells is due to a pronounced downregulation of L-selectin observed in activated Pak1-deficient T cells compared to WT.

### Pak1/JNK/Foxo1 Pathway Regulates L-selectin and CCR7 Gene Transcription

**Figure 3:**
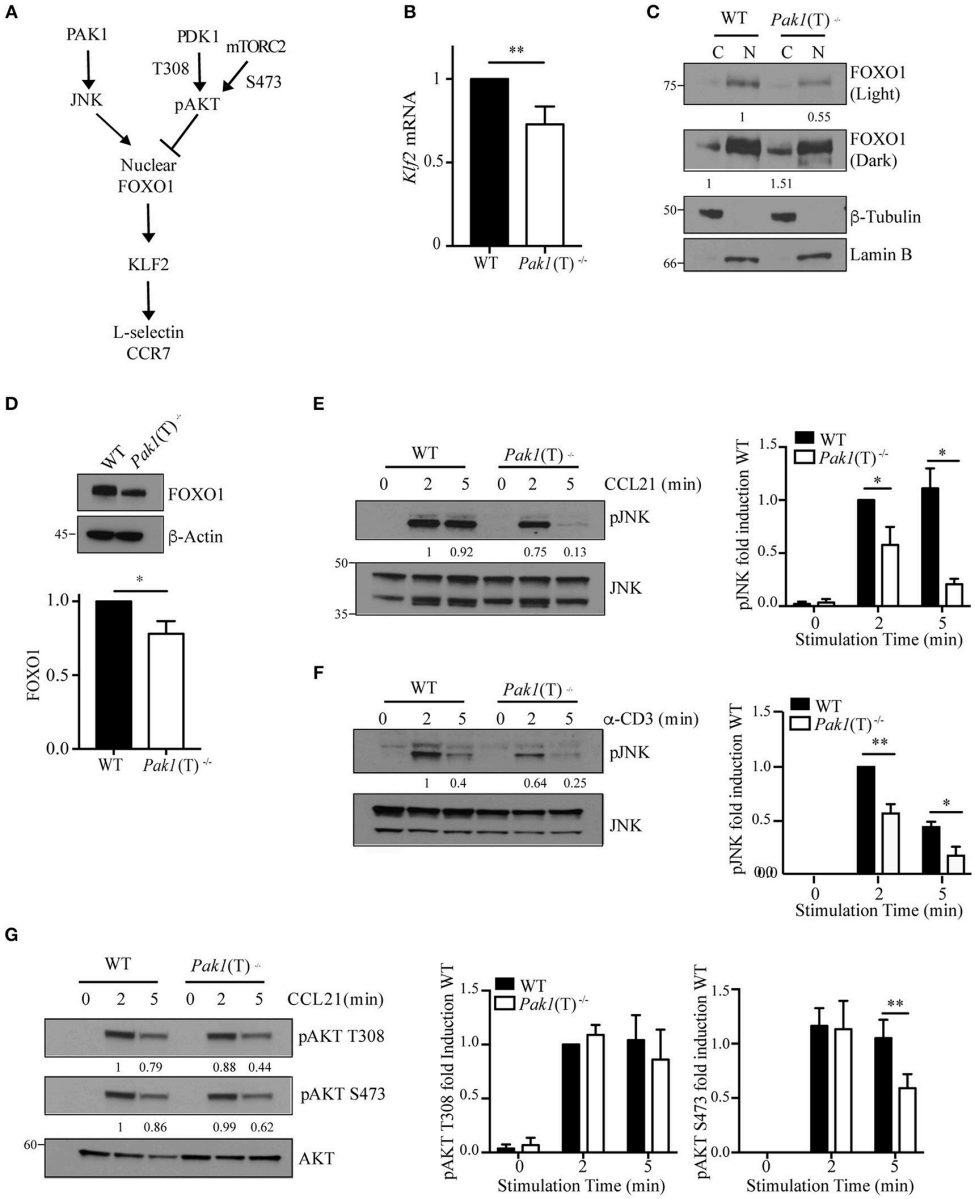
Regulation of Foxo1/Klf2 pathway by Pak1/JNK. **(A)** Scheme of Foxo1 nuclear localization regulation. **(B)** Relative expression of *Klf2* mRNA by qRT-PCR in CD4^+^ T cell blasts from WT and *Pak1*(T)^−/−^ mice. Data were pooled from five independent experiments. **(C)** Lamin-B nuclear and tubulin-enriched cytosolic fractions were prepared from WT and *Pak1*(T)^−/−^ CD4^+^ T blasts. Foxo1 nuclear translocation was studied by western-blot. Foxo1 levels in cytosol or nucleus were normalized to tubulin or lamin B, respectively. Numbers indicate the relative abundance of Foxo1 in comparison to WT cells. Results from one of more than three independent experiments are shown. **(D)** Lysates from WT and *Pak1*(T)^−/−^ CD4^+^ T blasts were analyzed with the indicated antibodies to measure total Foxo1; bottom, abundance of Foxo1 relative to that of β-actin pooled from five independent experiments. **(E)** WT and *Pak1*(T)^−/−^ CD4^+^ T blasts were activated with CCL21 (200 ng/mL) **(F)** or anti-CD3 for the indicated times and lysed. Lysates were then analyzed with the indicated antibodies to measure JNK phosphorylation. Numbers indicate the relative abundance of normalized pJNK to JNK, in comparison to WT cells. The bar graph shows the quantification of these measurements. Results from one of three independent experiments are shown. **(G)** WT and *Pak1*(T)^−/−^ CD4^+^ T blasts were activated with CCL21 (200 ng/mL) for the indicated times and lysed. Lysates were then analyzed with the indicated antibodies to measure AKT phosphorylation. AKT phosphorylation levels were normalized to AKT. Numbers indicate the relative abundance of pAKT T308 and S473 in comparison to WT cells. The bar graph shows the quantification of these measurements. Results from one of five independent experiments are shown. Statistical analysis: unpaired Student's *t*-test **(B,D–G)**. *ns*, not significant; *P < 0.05; **P < 0.01.

The transcription factor Klf2 regulates the expression of L-selectin and CCR7 (Figure 3A). We examined *Klf2* mRNA expression and observed a decrease in *Klf2* gene transcription in *Pak1*(T)^−/−^ CD4^+^ and CD8^+^ T cell blasts compared to WT T cells (Figure 3B, Figure S2B). Klf2 transcription, in turn, is regulated by the transcription factor, Foxo1 (28). In naïve T cells Foxo1 is transcriptionally active in the nucleus. Following T cell activation, Foxo1 is phosphorylated, driving it from the nucleus to the cytosol where it forms a complex with a 14-3-3 binding protein, thereby terminating transcription of Klf2 and its gene targets (28). Because nuclear Foxo1 levels regulate Klf2, L-selectin, and CCR7 expression (29), we measured the amount of Foxo1 protein in the cytosolic and nuclear cellular fractions of WT and *Pak1*(T)^−/−^ CD4^+^ T cell blasts. Levels of Foxo1 were higher in the nucleus compared with the cytosolic fraction in both WT and *Pak1*(T)^−/−^ CD4^+^ T cell blasts. However, in the absence of Pak1 our results showed a reduction in the levels of Foxo1 in the nucleus and an increase in the cytosol in comparison with WT lysates (Figure 3C). Moreover, the total levels of Foxo1 in *Pak1*(T)^−/−^ CD4^+^ T cell blasts were reduced compared with WT CD4^+^ T cell blasts (Figure 3D). Our results are consistent with the observation that Klf2, L-selectin and CCR7 expression was decreased in Foxo1-null T cells (29).

As JNK and AKT kinases regulate FOXO1 activation in opposite ways (30), we studied the level of activation of both kinases. We measured JNK phosphorylation and observed a dramatic decrease in JNK activation upon CCR7 or TCR stimulation in *Pak1*(T)^−/−^ CD4^+^ T cell blasts, while JNK activation was sustained in WT cells (Figures 3E,F). A decrease in JNK activation would lead to less Foxo1 phosphorylation and nuclear translocation, making more Foxo1 available in the cytoplasm for AKT phosphorylation and binding of 14-3-3. In support of this result we have reported previously that Pak1 activity increases JNK activation in primary and in Jurkat T cells (13). We also studied the status of AKT activation, as a negative regulator of FOXO1 activation and an indicator of PI3K and mTOR signaling. PI3K through PDK1 regulates the phosphorylation of AKT at Thr308, while AKT phosphorylation at Ser473 is regulated by mTORC2 (31, 32). While no differences were observed in the phosphorylation of AKT at Thr308, a slight decrease in AKT activation at Ser473 after 5 min of stimulation with CCL21 was detected in *Pak1*(T)^−/−^ compared to WT CD4^+^ T cell blasts (Figure 3G). Thus, though the cause for the decrease in AKT phosphorylation in Pak1-deficient T cells is unclear, Foxo1 localization to the nucleus appears to be regulated primarily by the JNK pathway (Figure 3C).

Collectively, these data indicate that Pak1, by activating JNK, which phosphorylates Foxo1 to promote its nuclear localization, positively regulates the gene transcription of the homing receptors L-selectin and CCR7.

### Pak1 Regulates Calcium-Dependent Shedding of L-selectin in Activated CD4^+^ T Cells

**Figure 4:**
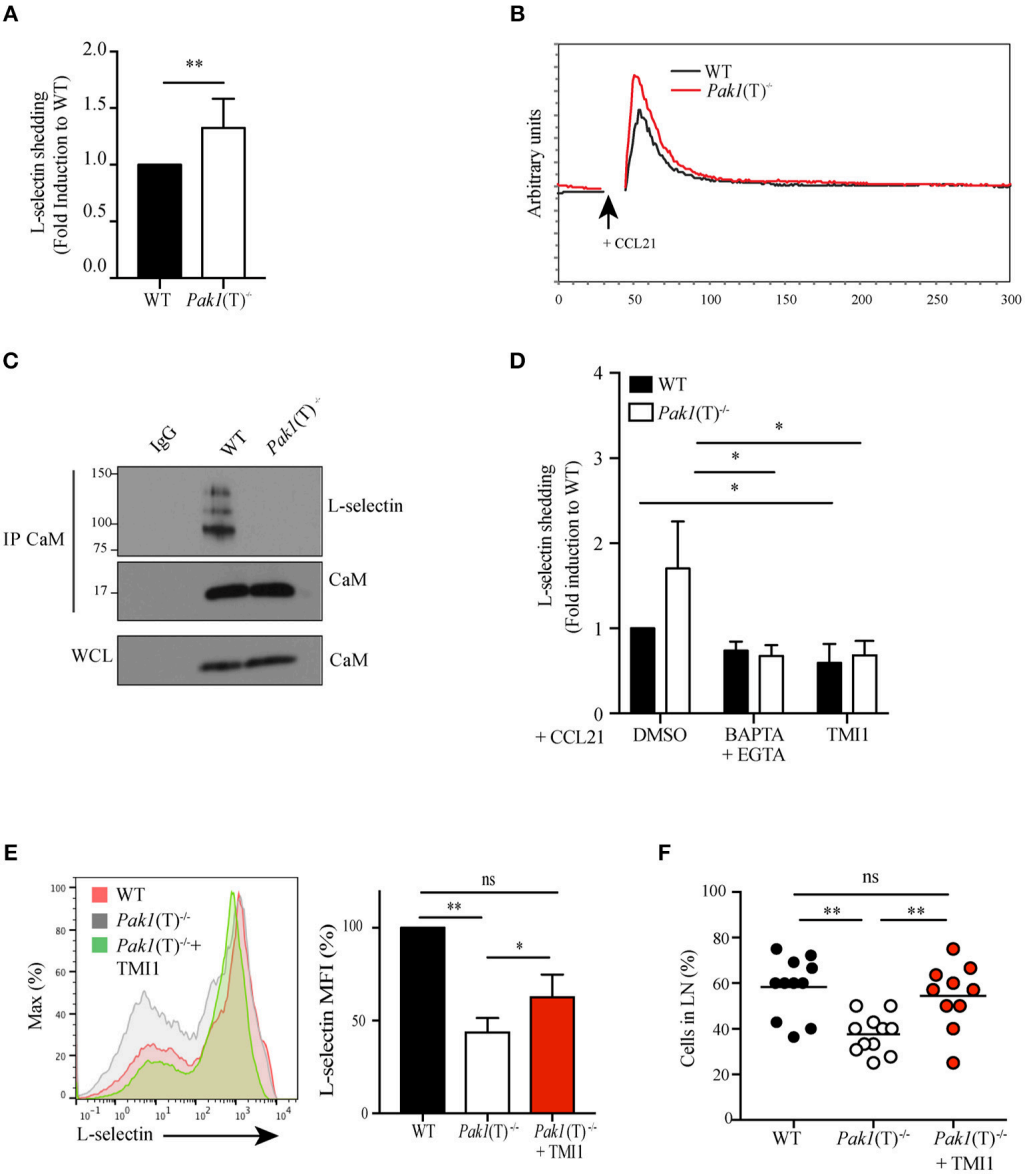
Pak1 regulates L-selectin shedding in activated CD4^+^ T cells. **(A)** Analysis by ELISA of soluble L-selectin in the supernatants of WT and *Pak1*(T)^−/−^ T cell blasts. Data were pooled from six independent experiments. **(B)** Representative time-dependent calcium flux (indo-violet/indo-blue) of Indo-1AM loaded WT and *Pak1*(T)^−/−^ CD4^+^ T cell blasts, stimulated with CCL21 (200 ng/mL) for the indicated time. Results from one of three independent experiments are shown. **(C)** Calmodulin (CaM) was immunoprecipitated and eluted material was blotted for co-precipitation L-selectin and CaM in lysates prepared from WT and *Pak1*(T)^−/−^ T cell blasts. Whole-cell lysates were blotted for CaM. Results from two independent experiments are shown. **(D)** Analysis of soluble L-selectin in the supernatant of WT and *Pak1*(T)^−/−^ CD4^+^ T cell blasts pre-treated for 20 min with DMSO (1:1,000), BAPTA-AM 10 μM and EGTA 2 mM, or TMI1 (0.5 μM), and incubated for 30 min in the presence of 100 ng/mL CCL21. **(E)** Left, L-selectin surface marker expression as measured by flow cytometry from WT and *Pak1*(T)^−/−^ CD4^+^ T cell blasts cultured with or without TMI1(10 μM) for 6 days. The results are representative of four experiments. Right, bar chart shows MFI (%) of L-selectin in WT and *Pak1*(T)^−/−^ blast T cells. **(F)** Percentage of WT, *Pak1*(T)^−/−^ and *Pak1*(T)^−/−^ treated with TMI1 cells per field of view. Statistical analysis: unpaired Student's *t*-test **(A)**; ANOVA between selected columns with Sidak's multiple comparison test **(D)**; ANOVA with Tukey's multiple comparison test **(E,F)**. *ns*, not significant; *P < 0.05; **P < 0.01.

Surface expression of L-selectin is also regulated by proteolytic cleavage mediated by the ADAM metalloproteinase 17, also known as tumor necrosis factor (TNF)-converting enzyme (TACE). A constitutive basal cleavage of L-selectin is observed in resting T cells but upon leukocyte activation via chemokine receptors or the TCR complex, the extracellular domains of L-selectin are rapidly cleaved at a membrane-proximal cut site by ADAM-17 (33, 34). To define whether Pak1 regulates the proteolytic cleavage and shedding of L-selectin we used an ELISA assay to compare the amount of the soluble cleavage product of L-selectin in the culture supernatants of activated WT and *Pak1*(T)^−/−^ T cells. Higher levels of soluble L-selectin were found in the supernatant of *Pak1*(T)^−/−^ CD4^+^ (Figure 4A) and CD8^+^ (Figure S2C) T cell blasts compared with WT T cells, corresponding to a decrease in L-selectin surface expression (Figure 2A; Figure S2A).

Calmodulin binds to the transmembrane domain of L-selectin and pulls the cleavage site closer to the plasma membrane, thus protecting it from being shed (35). T cell activation generates a local increase in calcium concentration that results in release of calmodulin from L-selectin due to the increased affinity of calmodulin to calcium. We studied the status of calcium influx after CCR7 activation and we observed an increase in calcium release in *Pak1*(T)^−/−^ CD4^+^ (Figure 4B) and CD8^+^ (Figure S2D) T cell blasts compared with WT. In support of this result we previous demonstrated that overexpression of Bam32 or Pak1 resulted in more non-phosphorylated PLC-γ1 and less calcium influx in T cells (12). The elevated calcium influx in activated *Pak1*(T)^−/−^ T cells suggested a possible reduction in the calmodulin binding to L-selectin. To test this notion, we measured calmodulin-L-selectin binding by a co-immunoprecipitation assay, using a calmodulin antibody for precipitation and a L-selectin antibody that recognizes the extracellular domain of the adhesion molecule to detect the un-cleaved total L-selectin bound to calmodulin. Consistent with our prediction, we observed a reduction in calmodulin binding to L-selectin in activated *Pak1*(T)^−/−^ CD4^+^ T cells (Figure 4C). Next, we tested if calcium influx directly regulated L-selectin shedding on the surface of activated *Pak1*(T)^−/−^ T cells. Toward this, we treated WT and *Pak1*(T)^−/−^ CD4^+^ T cell blasts with BAPTA-AM plus EGTA, to prevent intracellular calcium elevation, TMI1, an ADAM17 inhibitor (36) or as a control DMSO. Upon treatment with BAPTA-AM and EGTA, we observed a reversal in the increased shedding of L-selectin observed in *Pak1*(T)^−/−^ CD4^+^ or CD8^+^ T cell blasts, which was not the case with the WT T cells. Our studies with ADAM17 inhibitor TMI1, also confirmed that ADAM17 is the predominant regulator of L-selectin shedding in the T cells when stimulated with CCL21 (Figure 4D, Figure S2E). Our results suggest that Pak1 controls L-selectin proteolytic cleavage and shedding by affecting calcium release.

Altogether, our results indicate that the decrease in L-selectin at the surface of Pak1-deficient T cells is multifactorial. Multiple transcription factors, which control L-selectin levels are decreased. Additionally, levels are affected by increased L-selectin cleavage and shedding, due to the decreased binding of calmodulin to the cytoplasmic tail of L-selectin.

### Increase in L-selectin Expression in Pak1-deficient T Cells Rescues Homing to Lymph Nodes

To test the hypothesis that the defect in T cell trafficking to lymph nodes observed in *Pak1*(T)^−/−^ T is a consequence of reduced L-selectin levels, we attempted to increase the levels of L-selectin at the membrane of *Pak1*(T)^−/−^ CD4^+^ T cell blasts using two different approaches. First, we treated effector T cells with PI3K (LY294002) or mTOR (rapamycin) inhibitors, which are known to promote the expression of L-selectin at the plasma membrane by enhancing its transcription (34). Consistent with the literature, the levels of L-selectin were increased in WT or *Pak1*(T)^−/−^ T cells blasts treated with either Ly294002 or rapamycin compared with cells treated with DMSO (Figure S3A). *Pak1*(T)^−/−^ CD4^+^ T cell blasts treated with rapamycin were used to study their capacity to home to lymph nodes (Figure S3B). Importantly, rapamycin-treated *Pak1*(T)^−/−^ CD4^+^ T cell blasts migrated to the lymph nodes comparable to the WT controls, while we observed a significant defect in migration properties of untreated *Pak1*(T)^−/−^ T cell blasts. Non-significant reduction in the percentage of untreated *Pak1*(T)^−/−^ T cells that migrate to spleen as well as untreated *Pak1*(T)^−/−^ T cells remain in the blood, was observed when compared with rapamycin-treated *Pak1*(T)^−/−^ or WT T cell blasts (Figure S3C). Next, to block shedding, we used TMI-1 to inhibit ADAM17 and restored the surface amounts of L-selectin in *Pak1*(T)^−/−^ CD4^+^ T cell blasts similar to WT controls (Figure 4E). By 2-photon intravital microscopy of the inguinal node we observed a significant rescue in the ability of CD4^+^ T cell blasts treated with TMI-1 to home to lymph nodes, when compare with untreated *Pak1*(T)^−/−^ or WT T cell blasts (Figure 4F; Videos S5–S7). Overall, by enhancing expression of L-selectin on the surface of *Pak1*(T)^−/−^ blast T cells, we restored trafficking of Pak1-deficient T cells to peripheral lymph nodes.

## Discussion

In this study we define the importance of Pak1 in regulating T cell trafficking to peripheral lymph nodes. We show that this enzyme regulates the expression of the lymph-node homing receptors, L-selectin and CCR7, at the surface of activated T cells. Levels of L-selectin and CCR7 in Pak1-deficient compared to WT T cells were lower as measured by protein and mRNA levels. Several factors accounted for these decreases. The levels of the transcription factors Foxo1 and Klf2, which induce the transcription of L-selectin and CCR7, were lower in *Pak1*(T)^−/−^ T cells. In addition, we also found that lack of Pak1 led an increase in the shedding of L-selectin in Pak1-deficient T cells which correlated with the recruitment of calmodulin to the cytoplasmic tail of L-selectin. Thus, our data provide evidence for two independent pathways downstream of Pak1 in T cells that converge to regulate the expression of lymph-node homing receptors (Figure S3D).

We find that Pak1-deficient T cells exhibit prominent defects in *in vivo* homing to lymph nodes. However, the motility of those cells that reach the lymph node was normal. To determine the mechanism of the trafficking defect into peripheral lymph nodes of activated, but not naïve Pak1-deficient T cells, we analyzed the expression of three crucial molecules, L-selectin, CCR7 and LFA-1, which regulate the entry of lymphocyte into the lymph nodes (2). We found that the surface expression of the leukocyte selectin L-selectin and the chemokine receptor CCR7 was decreased in activated *Pak1*(T)^−/−^ T cells, whereas LFA-1 expression was not affected. Modification of L-selectin and CCR7 cell surface expression can change lymphocyte recirculation patterns and have an impact on immune responses (37–39). The transcriptional downregulation of the lymph node homing molecules, L-selectin and CCR7, after immune activation prevents effector T cells from re-entering peripheral lymph nodes and favors their migration to peripheral tissues where they exert their effector function (4, 37). Consistent with the literature, we found that a deficiency in L-selectin and CCR7 do not affect homing to the spleen (25–27).

Pak1 deficiency had an effect on the transcriptional mechanisms that down-modulate L-selectin and CCR7 expression in activated T cells, as we observed a decrease in the mRNA levels of CCR7 and L-selectin, and in the L-selectin transcription factor Klf2. The transcription factor FOXO1 binds to the KLF2 promoter in T cells (28) and Foxo1-deficient T cells decrease L-selectin expression (29). Also, FOXO1 induces CCR7 mRNA expression in Jurkat T cells (28), whereas *Klf2* deficiency regulates surface expression of CCR7 but not its transcription (40). We observed an increase in cytoplasmic Foxo1 in the absence of Pak1, in addition to a decrease in the total amount of Foxo1. FOXO transcription factors are closely regulated by post-translational modifications that affect their protein levels, localization and activity (41, 42). They undergo inhibitory phosphorylation by protein kinases such as AKT, driving FOXO1 from the nucleus to the cytosol and inducing binding of cytoplasmic chaperones 14-3-3 to FOXO1 (51). By contrast, FOXO1 is activated by upstream regulators such as JNK (30). JNK phosphorylates FOXO1 preventing interaction of 14-3-3 scaffold proteins with FOXO1, thereby promoting FOXO1 nuclear localization. Previous studies showed that PI3K signaling inhibits the expression of Klf2, L-selectin and CCR7 during T cell activation (21, 34). We observed a decrease in pAKT S473 activation in *Pak1* (T)^−/−^ T cell blasts; however, we observed a greater decrease in JNK phosphorylation in the absence of Pak1. Previous work from our laboratory demonstrated that Pak1 activation increases JNK activation in primary and in Jurkat T cells (13). Although the cause of the decrease in AKT phosphorylation in Pak1-deficient T cells is unclear, the decrease in JNK phosphorylation appears to be more important in regulating FOXO1 localization in this setting. Together, these data indicate that Pak1, by activating JNK, which phosphorylates Foxo1 to promote its nuclear localization, positively regulates the gene transcription of L-selectin and CCR7, and T cell lymph node homing.

The action of metalloproteinases regulate the surface expression of L-selectin (43). After cleavage, soluble L-selectin (sL-selectin) levels increase in the supernatant of activated T cells. By ELISA, we found high levels of sL-selectin in the supernatant of activated *Pak1*(T)^−/−^ T cells when compared to activated WT T cells. Leucocyte trafficking is affected by the capacity of sL-selectin to bind ligand and compete with cell-associated L-selectin. In addition, blocking L-selectin cleavage on T cells perturbs their migration and inhibits antiviral T cell responses (44–46). Calmodulin constitutively binds the cytoplasmic tail of L-selectin, and it is removed after an increase in calcium influx upon GPCR or immunoreceptor engagement, leading to L-selectin shedding (35). Using a co-immunoprecipitation experiment we observed a decrease in the binding of calmodulin to L-selectin in Pak1-deficient T cells. Consequent to Pak1 deficiency in T cells, we found an increase in calcium flux after CCR7 activation. Previously, we described that Bam32 or Pak1 overexpression decreases calcium influx after TCR engagement (12). Additionally, we observed that by blocking calcium flux or ADAM17 activity, sL-selectin levels in *Pak1*(T)^−/−^ T cells decrease. Together, these data support a model in which Pak1 regulates L-selectin amounts at two different levels, both transcriptional and membrane shedding, with the later regulated in a calcium-dependent manner.

As Foxo1 regulates CD4^+^ T cell differentiation, including the development and function of regulatory T cells (47–49), it would be interesting to study the development of induced T regulatory cells in *Pak1*(T)^−/−^ mice. Moreover, our work suggests that further studies of Pak1 in T cells could identify therapeutically useful ways to more selectively modulate L-selectin activity and activated T cell trafficking. Because of the increase in vascular L-selectin ligands in some diseases, such as chronic inflammation, acute dermatitis, rheumatoid arthritis, diabetes, and asthma (50), it would be interesting to test *Pak1*(T)^−/−^ mice for disease susceptibility in these different conditions.

## Author Contributions

AD-E, NM, BS, RW, and LS designed experiments. AD-E performed experiments, analyzed the data, and generated figures. AD-E and NM performed 2-photon intravital experiments under the supervision of RW. LS supervised experiments and analysis. AD-E and LS wrote the manuscript with contributions from the other authors.

### Conflict of Interest Statement

The authors declare that the research was conducted in the absence of any commercial or financial relationships that could be construed as a potential conflict of interest.
