# Supplementary material for: Pak1 Kinase Promotes Activated T Cell Trafficking by Regulating the Expression of L-Selectin and CCR7
Source: Front Immunol. 2019 Mar 5;10:370. doi: 10.3389/fimmu.2019.00370 (PMC6411651; doi:10.3389/fimmu.2019.00370)
Supplement: Supplementary file 8 [file Data_Sheet_1.docx]

*Supplementary Materials*

**Pak1 kinase promotes activated T cell trafficking by regulating the expression of L-selectin and CCR7**

**Ana Dios-Esponera, Nicolas Melis, Bhagawat C. Subramanian, Roberto Weigert and Lawrence E. Samelson^*^**

^*^**Correspondence:** Dr. Lawrence E Samelson: [samelsonl@helix.nih.gov](mailto:samelsonl@helix.nih.gov)

^^

**Figure S1. Pak1-depletion does not affect naïve CD4^+^ T cell migration or the motility of CD4^+^ T cell blasts inside the inguinal node.** Differentially dye-labeled splenocytes **(A)** CD4^+^ WT and *Pak1*(T)^-/-^ T cells were co-adoptively transferred intravenously at a 1:1 ratio and T cell trafficking to lymphoid tissues was quantified by flow cytometry. Recovery of co-transferred labeled WT or *Pak1*(T)^-/-^ cells, presented as a percentage of total transferred cells recovered from the blood, LNs or spleen at 1 h after transfer. Each dot indicates results from an individual mouse; horizontal bars indicate the mean. Data are pooled from four recipient mice of two independent experiments. Sample sizes: 8, blood and spleen and 7 lymph nodes. **(B)** Statistical analysis of track duration from translocated cells. Each symbol represents an individual cell (10 WT, 9 *Pak1*(T)^-/-^). **(C-E)** Quantification of 2-photon intravital microscopy motility parameters pooled from image sequences in independent experiments. Mean track speeds (C), arrest coefficient (B), meandering index (E) of WT and *Pak1*(T)^-/-^ T cell blasts in LN. Each symbol represents an individual cell (154 WT, 94 *Pak1*(T)^-/-^). Statistical analysis: unpaired Student’s t test (A and B); Mann-Whitney test (C and D). *ns*, not significant; **P* < 0.05.

**Figure supplementary 2. Decreased expression of L-selectin in Pak1-deficient CD8^+^ activated T cells. (A)** Flow cytometry evaluation of L-selectin on blast T cells. Representative contour plots for three independent experiments showing L-selectin status comparison between WT and *Pak1*(T)^-/-^ are shown. Bar chart shows MFI of L-selectin in WT and *Pak1*(T)^-/-^ blast T cells. **(B)** Relative expression of *Klf2 and Sell* mRNA by qRT-PCR in blast CD8^+^ T cells from WT and *Pak1*(T)^-/-^ mice. Pooled from three independent experiments. **(C)** Analysis of soluble L-selectin in the supernatants of WT and *Pak1*(T)^-/-^ blast T cells from three independent experiments. **(D)** Representative Ca^2+^ flux as assessed by fluorometric evaluation after CCL21 stimulation of indicated CD8^+^ T cells. Kinetics of the ratio of indo-blue to indo-violet over time shown and assessed by flow cytometry. Results from one of three independent experiments are shown. **(E)** Analysis of soluble L-selectin in the supernatant of blast CD8^+^ T cells pre-treated for 20 min with DMSO (1:1000), BAPTA-AM 10µM and EGTA 2mM, or TMI1 (0.5 μM), and incubated for 30 min in the presence of 100 ng/mL CCL21. Statistical analysis: unpaired Student’s t test (B and C). *ns*, not significant; **P* < 0.05.

**Figure supplementary 3. Pak1 regulates L-selectin in activated CD4^+^ T cells. (A)** Representative contour plots for 3 independent experiments showing L-selectin surface expression on WT and *Pak1*(T)^-/-^ CD4^+^ T cell blasts cultured 48h in IL2 with or without DMSO (1:200), Ly294002 (10µM) and rapamycin (20nM). **(B)** L-selectin expression bar chart shows MFI (%) of L-selectin from four different experiments. **(C)** Blast CD4^+^ T cells from WT and *Pak1*(T)^-/-^ mice were either untreated or treated with rapamycin for 48 h, labeled with CMFDA, CTV and Far Red dyes, and mixed at a ratio of 1:1:1 prior to injection into C57BL/6 hosts. The data show the percentage of CD4^+^ T cells of the total number of transferred cells recovered in the blood, lymph nodes or spleen 1 h after transfer. Data are pooled from four to five recipient mice of three independent experiments. Sample sizes: 14, blood, lymph node and spleen. **(D)** Proposed pathways mediating Pak1 control of L-selectin expression in activated T cells. Gray arrows indicate relative action caused by Pak1 deficiency. Statistical analysis: unpaired Student’s t test (A); ANOVA with Tukey’s multiple comparison test (B and C). *ns*, not significant; **P* < 0.05; ** *P* < 0.01; ****P* < 0.001; *****P* < 0.0001.

Video S1. **2-photon intravital image sequence of WT and *Pak1*(T)^-/-^ CD4^+^ T cell blasts migration in the parenchyma of the inguinal node after 15 min of i.v. injection.** HEVs are labeled blue. Bar, 50 µm, time in min and s.

Video S2. **2-photon intravital image sequence of a WT CD4^+^ T cell blasts performing TEM in the inguinal node 15 min of i.v. injection.** HEVs are labeled blue. Bar, 50 µm, time in min and s.

Video S3. **2-photon intravital image sequence of a *Pak1*(T)^-/-^ [**[**32**](#_ENREF_32)**] CD4^+^ T cell blasts performing TEM in the inguinal node 15 min of i.v. injection.** HEVs are labeled blue. Bar, 50 µm, time in min and s.

Video S4. **2-photon intravital image sequence of WT and *Pak1*(T)^-/-^ CD4^+^ T cell blasts migration in the parenchyma of the inguinal node 1 h after i.v. injection.** HEVs are labeled blue. Bar, 50 µm, time in min and s.

Video S5. **2-photon intravital image sequence of untreated *Pak1*(T)^-/-^ and *Pak1*(T)^-/-^ CD4^+^ treated with TMI-1 [**[**51**](#_ENREF_51)**]T cell blasts migration in the parenchyma of the inguinal node.** HEVs are labeled blue. Bar, 50 µm, time in min and s.

Video S6. **2-photon intravital image sequence of untreated WT and *Pak1*(T)^-/-^ CD4^+^ treated with TMI-1 T cell blasts migration in the parenchyma of the inguinal node.** HEVs are labeled blue. Bar, 50 µm, time in min and s.

Video S7. **2-photon intravital image sequence of untreated WT and *Pak1*(T)^-/-^ CD4^+^ T cell blasts migration in the parenchyma of the inguinal node.** HEVs are labeled blue. Bar, 50 µm, time in min and s.
